# Counterintuitive Pseudoscience Propagates by Exploiting the Mind’s Communication Evaluation Mechanisms

**DOI:** 10.3389/fpsyg.2021.739070

**Published:** 2021-10-05

**Authors:** Spencer Mermelstein, Tamsin C. German

**Affiliations:** Department of Psychological and Brain Sciences, University of California, Santa Barbara, Santa Barbara, CA, United States

**Keywords:** epistemic vigilance, pseudoscience, counterintuitive concepts, memory, social transmission, astrology, parapsychology, epidemiology of representations

## Abstract

Epidemiological models of culture posit that the prevalence of a belief depends in part on the fit between that belief and intuitions generated by the mind’s reliably developing architecture. Application of such models to pseudoscience suggests that one route via which these beliefs gain widespread appeal stems from their compatibility with these intuitions. For example, anti-vaccination beliefs are readily adopted because they cohere with intuitions about the threat of contagion. However, other varieties of popular pseudoscience such as astrology and parapsychology contain content that violates intuitions held about objects and people. Here, we propose a pathway by which “counterintuitive pseudoscience” may spread and receive endorsement. Drawing on recent empirical evidence, we suggest that counterintuitive pseudoscience triggers the mind’s communication evaluation mechanisms. These mechanisms are hypothesized to quarantine epistemically-suspect information including counterintuitive pseudoscientific concepts. As a consequence, these beliefs may not immediately update conflicting intuitions and may be largely restricted from influencing behavior. Nonetheless, counterintuitive pseudoscientific concepts, when in combination with intuitively appealing content, may differentially draw attention and memory. People may also be motivated to seek further information about these concepts, including by asking others, in an attempt to reconcile them with prior beliefs. This in turn promotes the re-transmission of these ideas. We discuss how, during this information-search, support for counterintuitive pseudoscience may come from deference to apparently authoritative sources, reasoned arguments, and the functional outcomes of these beliefs. Ultimately, these factors promote the cultural success of counterintuitive pseudoscience but explicit endorsement of these concepts may not entail tacit commitment.

## Introduction

Pseudoscience—claims that take on the guise of scientific knowledge but lack evidentiary support or theoretical plausibility—is pervasive. At least 40% of Americans, for example, believe in extra-sensory perception and 25% believe that the position of the stars affects life on Earth (Moore, 2005). Pseudoscience can be harmful. The proliferation of anti-vaccination sentiments undermines public health campaigns (Larson et al., 2011) and misinformation about global climate change reduces support for mitigation efforts (van der Linden et al., 2017). Understanding the psychological appeal and social transmission of pseudoscience is therefore critical for informing attempts to reduce the impact and spread of these beliefs.

Blancke and De Smedt (2013), Boudry et al. (2015), and Blancke et al. (2017), Blancke et al. (2019) recently advanced a model accounting for the ubiquity of pseudoscience. Drawing on Sperber’s (1994,1996) epidemiological theory of cultural representations, these authors have suggested that many forms of pseudoscience are widespread because they cohere with intuitive ways of thinking. For example, those opposed to vaccination often point to pseudoscientific claims that vaccines might cause autism spectrum disorders or other harm (Poland and Spier, 2010). Miton and Mercier (2015) suggest that vaccines, as they entail injecting (inert) pathogens into the body, tap into disgust intuitions that evolved to protect against exposure to contaminants. Vaccines may then be intuitively viewed as a source of contagion, making anti-vaccination claims centered on harm inherently believable, appealing, and transmissible from mind to mind. Other pseudoscientific beliefs may gain traction by exploiting a variety of cognitive predispositions: Creationism/Intelligent Design is grounded in intuitive teleological reasoning (Kelemen, 2004; Blancke et al., 2017); anti-GMO attitudes are based in essentialist intuitions (Blancke et al., 2015); flat earth beliefs are rooted in naive mental models of a geocentric solar system (Vosniadou, 1994).

Along with pseudoscientific beliefs that might exploit a fit with intuitions, however, are a range of such beliefs that manage to spread despite content that is decidedly counterintuitive. Specifically, these “counterintuitive pseudoscientific” beliefs violate evolved and reliably developing core knowledge intuitions. Documented as early as infancy (Spelke and Kinzler, 2007), core knowledge intuitions structure our basic expectations of physical objects and their mechanics (e.g., Spelke, 1990) and of intentional agents and their mental states (Baillargeon et al., 2016), among other ontological domains. Thus, counterintuitive concepts are not merely unusual but rather are defined by their incompatibility with the foundational distinctions the mind makes in parsing the world. People may nonetheless acquire counterintuitive concepts; indeed, they are widespread throughout religious, scientific, and pseudoscientific belief systems (Boyer, 2001; Baumard and Boyer, 2013; Shtulman, 2017).

Astrology is one example of counterintuitive pseudoscience. Cultures as diverse as the Babylonians, Han Dynasty China, and the Maya each developed sophisticated belief systems and mathematics to divine the purported influence of the planets and stars on people’s personalities and events on Earth (Boxer, 2020). Moreover, astrology remains widespread today despite its contemporary status as a pseudoscience. This is true despite the fact that a central tenet of astrology, that celestial objects can have an influence on people or events on Earth, violates core “folk physics” intuitions that objects cannot act on each other at a distance (Leslie and Keeble, 1987; Spelke, 1990).

Parapsychology, or *psi*, is a second example of counterintuitive pseudoscience. The belief that psychics, mediums, and clairvoyants have a preternatural ability to read minds, manipulate or view distant objects, or tell the future has ancient roots in cultures around the world (Singh, 2018) and has been the subject of research for over 150 years despite its fundamental disconnect from the sciences (Reber and Alcock, 2020). Again, this is despite the fact that these beliefs violate core “folk psychological” intuitions that a person’s beliefs are constrained by their perceptual capacities: that people are ignorant of events they haven’t seen or heard (Onishi and Baillargeon, 2005; Baillargeon et al., 2016).

The ubiquity of pseudoscience that contains such drastically counterintuitive elements is potentially surprising from a cultural epidemiology perspective. One reason follows from the suggestion that the prevalence of a belief in a population may depend in part on its fit with intuitive ways of thinking (Sperber, 1994, 1996). On this account, information that is consistent with intuitions is generally more likely to persist across repeated retellings and become more widespread than counterintuitive information (Kalish et al., 2007; Griffiths et al., 2008; Morin, 2013; Miton et al., 2015).

A second potential obstacle to the spread of counterintuitive content stems from the suggestion that the mind contains a host of mechanisms designed to evaluate and filter communicated information (Sperber et al., 2010; see also Mercier, 2017). One function of these “epistemic vigilance” mechanisms is to assess the plausibility of a message by checking its consistency with prior beliefs. The rudiments of these consistency-checking mechanisms have been documented as early as infancy (Koenig and Echols, 2003), and by age 4, children have been found to reject the claims of others that conflict with their firsthand experiences (Clément et al., 2004) or background knowledge about objects and animals (Lane and Harris, 2015). Counterintuitive information, then, appears to be at a social transmission and believability disadvantage relative to information consistent with cognitive predispositions (Mercier et al., 2019). What then accounts for the cultural success of pseudosciences like astrology and parapsychology?

In the current article, we propose a pathway by which counterintuitive pseudoscience may spread and receive broad endorsement. First we suggest that these beliefs engage the mind’s communication evaluation mechanisms, which largely restrict their influence on behavior. Nonetheless, counterintuitive pseudoscience, as it cannot be fully reconciled with past beliefs, recruits our attention and memory, and triggers a search for more information that may result in the preferential re-transmission of these ideas. During information-search, endorsement of counterintuitive pseudoscience may be bolstered by support from apparently authoritative sources, reasoned arguments, or the functional outcomes of holding such beliefs. Counterintuitive pseudoscience thus achieves cultural prominence by exploiting the mind’s communication evaluation mechanisms but explicit belief in such content may not entail tacit commitment.

## The Representational Format of Counterintuitive Pseudoscience

While communication that is consistent with prior beliefs may be readily accepted, counterintuitive pseudoscience is a class of content that should be flagged by epistemic vigilance mechanisms as requiring further monitoring. By hypothesis, inconsistencies between counterintuitive content and pre-existing beliefs trigger epistemic vigilance mechanisms to quarantine that content from those beliefs via a “meta-representational” formatting (Sperber, 1997, 2000; see also Mercier, 2017).

A metarepresentation is a mental data structure that links a proposition to a set of tags that limit the scope of applicability of the information (Cosmides and Tooby, 2000). These tags may take the form of a link to a particular source (Mermelstein et al., 2020), a propositional attitude like certainty or doubt (Leslie, 1987), or a supporting argument (Mercier and Sperber, 2011). For example, the proposition “the stars influence events on earth” may be embedded in the metarepresentation “my friends believe that [the stars influence events on earth].” Encapsulated within contextualizing tags, counterintuitive concepts are prevented from spontaneously updating or interacting with existing beliefs or influencing behavior. Nonetheless, one may still come to explicitly profess belief in counterintuitive concepts, deliberately derive inferences from them, and articulate them to others—but only upon reflection as they cannot be reconciled with conflicting core intuitions (Sperber, 1997).

Epistemic vigilance mechanisms tend to quarantine, rather than outright reject, counterintuitive pseudoscientific beliefs like astrology and parapsychology for two reasons. First, such messages may often be communicated by friends, family, or other influential people. Epistemic vigilance mechanisms are therefore likely to retain these messages (albeit as metarepresentations), given underlying trust in these sources (Sperber, 1997; Sperber et al., 2010; Harris et al., 2018) and social learning biases that motivate people to adopt the beliefs of the successful or prestigious (Henrich and Gil-White, 2001). Relatedly, should a particular counterintuitive concept be widespread in a community, people might at least outwardly endorse such beliefs given that the social cost of rejecting a belief held by their peers may be greater than epistemic costs of harboring them (Hong and Henrich, 2021). Second, epistemic vigilance mechanisms might retain these concepts to aid in the further evaluation of their source and content over time (Mermelstein et al., 2020). Should we later come across information that supports or challenges a given claim, we can then update our judgment of the veracity of the message and the trustworthiness and/or competence of its speaker. Until corroborating evidence is found, we would expect counterintuitive pseudoscientific concepts to remain quarantined as reflectively-held metarepresentations, with consequences for their stability and capacity to influence behavior.

As reflectively-held beliefs, the counterintuitive concepts found in some varieties of pseudoscience may be variable in their specific content (Baumard and Boyer, 2013). Whereas intuition-consistent pseudoscience might coalesce around a small set of cognitively appealing claims (e.g., “vaccine ingredients cause harm”), counterintuitive beliefs such as “psychics know the future” may be subject to differing and possibly idiosyncratic interpretations. Compatible with this suggestion, proponents of *psi* have put forward a wide range of different accounts for the underlying mechanisms through which these abilities work (Reber and Alcock, 2020). Some accounts, for instance, reference paranormal forces (e.g., a connection to a spirit world), while others may (erroneously) implicate scientific explanations (e.g., quantum mechanics). Without grounding in intuition, the exact content of counterintuitive pseudoscience may be *ad hoc*; moreover, these beliefs may be inconsistent or contradictory even within the same mind, as has been documented among adherents of conspiracy theories (Wood et al., 2012) and religious beliefs (Slone, 2007).

Another proposed signature of reflectively-held beliefs is that they may coexist alongside the intuitions with which they conflict rather than update or replace them (Sperber, 1997). Indeed, representational co-existence has been documented for counterintuitive concepts found in science (Kelemen and Rosset, 2009; Shtulman and Valcarcel, 2012; Shtulman and Harrington, 2016) and religion (Barrett and Keil, 1996; Barrett, 1998; Barlev et al., 2017, 2018, 2019). Research on the God concept, for example, finds that religious believers accurately describe God’s counterintuitive properties (e.g., omnipresence, omniscience) when explicitly asked, but nonetheless reason as though God possessed human-like psychology and physicality when indexed by implicit measures (Barrett and Keil, 1996; Barrett, 1998). Co-existence also raises the possibility of interference between mutually incompatible beliefs. Barlev et al. (2017, 2018, 2019) asked religious believers to evaluate a series of statements that were consistent or inconsistent in truth-value between intuitions about persons and later-acquired counterintuitive beliefs about God. Participants were slower and less accurate at evaluating inconsistent vs. consistent statements, suggesting that intuitions not only co-exist alongside incompatible beliefs, but also conflict with them. The ongoing tension between core intuitions and counterintuitive concepts suggests that these beliefs, including those found in pseudoscience, may not regularly inform behavior.

An implication of this idea is that counterintuitive pseudoscientific concepts might only be deployed in narrow contexts, giving rise to discrepancies between stated beliefs and everyday behavior (Sperber, 1985; Barrett, 1999; Slone, 2007). While one might state their belief that a psychic can tell the future or even follow their horoscope’s recommendations when making decisions, they might do so only upon reflection or when prompted. Commitment to counterintuitive pseudoscientific beliefs might generally be at a reflective and not an intuitive level. Indeed, such beliefs may be largely decoupled from behavior as a function of epistemic vigilance mechanisms. A typical believer in *psi*, for instance, would likely make quite different decisions in their life should they implicitly believe that someone could be watching them at any time; the position of the stars and planets may not be one’s initial explanation for another’s behavior but a *post hoc* rationalization. In contrast, intuition-consistent pseudoscience may have a more direct influence on behavior. Unencumbered by a metarepresentational formatting, anti-vaccination beliefs, for instance, might fluidly translate to vaccine refusal (Miton and Mercier, 2015).

## Memory for Counterintuitive Pseudoscience

The memorability of a concept is one predictor of its cultural success: memorable content, all things equal, is more likely to be reproducible and retain fidelity across retellings. Past research suggests that a subset of counterintuitive pseudoscientific beliefs may be mnemonically optimal. Boyer (1994, 2001, 2003) has argued that concepts which are largely consistent with the expectations afforded to ontological categories such as “person” or “object” but for a minimal set of violations of those expectations are particularly attention-grabbing, memorable, and inferentially rich. The concept of a ghost fits this “minimally counterintuitive” template: despite being deceased and capable of passing through solid objects, ghosts are otherwise conceptualized as persons with beliefs and desires. Such striking violations of expectations draw attention as they cannot be fully incorporated into existing beliefs, yet we may still easily imagine and make inferences about ghosts using our knowledge about people. Together, these features make for a differentially memorable combination compared to fully ordinary concepts. Counterintuitive concepts with many violations of expectation (e.g., “a ghost that knows nothing and could never interact with the world”), however, lose their memorability advantage as they cease to hook into existing knowledge and fail to yield many meaningful inferences.

Boyer’s (2001) account has received empirical support from laboratory experiments with adults from across cultures (e.g., Boyer and Ramble, 2001; Nyhof and Barrett, 2001) and with children (Banerjee et al., 2013). Participants in these studies were asked to recall or retell narratives to others, with results demonstrating a memory advantage for minimally counterintuitive (e.g., “a chair that can float in midair”) compared to ordinary (e.g., “a table that can hold a lot of weight”) or very counterintuitive concepts (e.g., “a rock that could give birth to a singing teapot”). The memory advantage for minimally counterintuitive concepts has also been found to extend to the contextual details associated with them, such as their speaker (Mermelstein et al., 2020). Furthermore, analyses of cultural materials such as folktales from around the world reveal that narratives containing minimally counterintuitive concepts tend to be more common than other concept types (Norenzayan et al., 2006; Burdett et al., 2009). Thus, the mind’s attention and memory mechanisms constrain the range of counterintuitive concepts that are likely to be remembered and suitable for cultural success.

We find it likely that popular counterintuitive pseudoscientific beliefs are composed of intuitive content alongside compelling, but limited violations of expectation. The wide range of *psi* abilities, for example, seem to be relatively narrow modifications of the capacities typically assumed of persons: supernatural mind-reading may be an overextension of everyday mentalizing, telekinesis an overextension of the expectation that mental states can have effects on the world by directing behavior. Psychics and the like, however, are otherwise conceptualized as ordinary people. The famous psychic Uri Geller could ostensibly bend spoons with his mind, but he nonetheless possessed a physical body that needed to eat, sleep, and breathe. Astrological belief systems may similarly package together counterintuitive and intuitive elements. While the claimed linkage between people and the position of the stars may violate intuitions of cause and effect, astrology does seem to feed off the human tendencies to perceive patterns in noise (Whitson and Galinsky, 2008), intuit purpose behind complex natural phenomenon (Kelemen et al., 2012), and stereotype others (Lu et al., 2020).

Future empirical work may investigate whether counterintuitive pseudoscientific content strikes a mnemonic optimum for cultural transmission. Laboratory studies employing serial re-transmission methods could demonstrate that minimally counterintuitive pseudoscientific concepts tend to survive repeated retellings compared to other content. Analyses of cultural materials could map out the degree to which astrologers or psychics draw upon counterintuitive vs. intuitively-appealing content in making their claims.

## Social Re-Transmission of Counterintuitive Pseudoscience

The prevalence of a belief in a population, however, depends not only on its memorability but also on individuals being willing to re-transmit it to others. Recent research suggests that people may share counterintuitive concepts with others in an attempt to gather more information about them. Indeed, as early as infancy, violations of expectation have been shown to trigger not only surprise but also information-seeking behavior: Stahl and Feigenson (2015) found that 11-month-old infants who saw an object involved in an counterintuitive event (e.g., a toy appeared to float in midair) preferentially explored that object and manipulated it in an attempt to learn more about its unusual properties in comparison to an ordinary object (e.g., one that fell when unsupported).

The early developing tendency to seek new information in response to a violation of core knowledge may extend across the lifespan, such that one may be motivated to learn more about counterintuitive concepts, including those found in pseudoscience, in an attempt to reconcile them with prior beliefs. One mode of information-search is to ask others for their opinion, thereby re-transmitting the concept. Compatible with this account, Mermelstein et al. (2019) found that novel counterintuitive statements (e.g., “a cactus that liked to sing”) were judged by adults to be less believable than ordinary statements (e.g., “a cat that liked to play with toys”), but also as more interesting, more desirable to learn about, and more likely to be passed along to others, and these variables were all strongly correlated. Thus, as with other epistemically suspect information (e.g., “fake news,” see Pennycook and Rand, 2021), one’s (lack of) belief in counterintuitive content seems to be orthogonal to a willingness to share it with others. People may repeat counterintuitive pseudoscience to others, regardless of their commitment to these beliefs, to scope out what others think about them.^1^

Nonetheless, Mercier et al. (2018) have put forward a complementary account suggesting that people may choose to re-transmit pseudoscientific beliefs so as to appear competent to others. Participants in this study rated a series of pseudoscientific (e.g., “people can learn information, like new languages, while asleep”) and factual (e.g., “handwriting doesn’t reveal personality traits”) statements on their believability, one’s willingness to re-transmit them, and on how knowledgeable someone who said that statement would seem. A key analysis found that the extent to which a participant believed that holding a given claim (pseudoscientific or factual) made them appear knowledgeable was an important predictor of their willingness to re-transmit it.

Mercier et al. (2018), however, did not differentiate intuition-consistent from counterintuitive pseudoscience. It may be the case that the motive for and method of re-transmission differs depending on the consistency of a claim with core intuitions. Thus, one may be willing to share, and desire to be associated with, intuition-consistent pseudoscience given that others might find that information intuitively compelling (Altay et al., 2020a). On the other hand, when re-sharing counterintuitive pseudoscientific beliefs one might tend to attribute them to a source other than the self while gauging others’ reactions to that content (Altay et al., 2020b). For example, disclaimers such as “I read somewhere that.” or “many other people have said.” allow one to discuss counterintuitive ideas with others without asserting ownership of them—all while promoting the circulation of counterintuitive pseudoscience from mind to mind.

## Discussion

In this article we have distinguished intuition-consistent from counterintuitive pseudoscience, described how the mind’s communication evaluation mechanisms might shape the representational characteristics of these counterintuitive concepts, and suggested how such beliefs may become memorable and socially transmissible. We speculate that, as people attempt to reconcile counterintuitive content with their prior beliefs, they may come across different lines of support that lead them to (at least explicitly) accept and endorse counterintuitive pseudoscience.

Mercier and Sperber (2011) have identified two ways by which communication that violates prior beliefs may overcome epistemic vigilance. First, one may suspend their disbelief in such information should they find its source(s) sufficiently trustworthy or reliable. Indeed, scientists have often come to counterintuitive conclusions (e.g., the sun is at the center of the solar system) and laypersons typically trust such claims based on the past reliability and esteem of science in general (Shtulman, 2013). Blancke et al. (2019) note that pseudoscience may become believable as it adopts the appearance of science and consequently its privileged epistemic status. Thus, when researchers publish apparent evidence of *psi* in peer reviewed journals, the public may be inclined to believe these claims have a degree of credibility given the source.

Second, acceptance of counterintuitive pseudoscience may come about by encountering supporting argumentation or reasons that justify holding these beliefs (Mercier and Sperber, 2011). Effective arguments in support of counterintuitive pseudoscience might emphasize links between that content and an audience’s cognitive predispositions or prior beliefs (Blancke et al., 2019), including existing commitments to other supernatural or paranormal beliefs (Lindeman and Aarnio, 2007). An astrologer’s predictions about the future might seem sensible in reference to intuitive pattern-seeking and teleological reasoning tendencies, a psychic might appeal to the widespread and cherished belief in spirits that survive the death of the body in explaining how they communicate with the deceased.

On that note, some strands of counterintuitive pseudoscience may be especially appealing, despite their inconsistency with core intuitions, as they function to alleviate stress or anxiety by providing a compensatory sense of control. Past research finds that many belief systems, from religious beliefs (Inzlicht and Tullett, 2010) and superstitious or magical thinking (Keinan, 2002) to belief in the efficacy of ritual behavior (Lang et al., 2015), may serve as a buffer against stressful or unpredictable circumstances by offering explanations and actions to take to reduce uncertainty or regain a sense of control (Kay et al., 2009). Interestingly, among pseudosciences, astrology and parapsychology have elements that might serve as anxiolytics. Indeed, experimental work has shown that participants induced to feel that outcomes were out of their control increasingly endorsed the existence of precognition (Greenaway et al., 2013) and followed a psychic’s recommendations (Case et al., 2004). Thus, certain counterintuitive pseudoscientific concepts may be particularly likely to gain acceptance, not because their content is intrinsically believable, but because of their functional role in reducing stress or anxiety.

## Conclusion

In conclusion, we have argued that counterintuitive pseudoscience has features that exploit the mind’s communication evaluation mechanisms to become attention-grabbing, memorable, and likely to be passed on to others. People may even come to explicitly endorse these beliefs through deference to an apparently authoritative source or from a reasoned argument. In this way, counterintuitive pseudoscience achieves cultural prominence. Nonetheless, we hypothesize that these beliefs are held reflectively as they cannot be reconciled with core intuitions (Sperber, 1997). As with other counterintuitive concepts in science (Shtulman and Valcarcel, 2012; Shtulman and Harrington, 2016) and religion (Barlev et al., 2017, 2018, 2019), such pseudoscientific beliefs may coexist alongside incompatible prior beliefs and may be to some extent suspended from guiding behavior. A stated belief in these concepts thus does not necessitate an implicit commitment to them in all contexts. Pseudoscience is ubiquitous but it is not unitary. Recognizing that these beliefs may propagate through different means may be key to undermining their spread and impact.

## Data Availability Statement

The original contributions presented in the study are included in the article/supplementary material, further inquiries can be directed to the corresponding author/s.

## Author Contributions

SM and TCG conceptualized the manuscript. SM wrote the first draft, to which TCG provided critical edits. Both authors contributed to its final version and approved its submission.

## Conflict of Interest

The authors declare that the research was conducted in the absence of any commercial or financial relationships that could be construed as a potential conflict of interest.

## Publisher’s Note

All claims expressed in this article are solely those of the authors and do not necessarily represent those of their affiliated organizations, or those of the publisher, the editors and the reviewers. Any product that may be evaluated in this article, or claim that may be made by its manufacturer, is not guaranteed or endorsed by the publisher.

## Footnotes

1 Interestingly, the philosopher David Hume (1748/2000) suggested that one may repeat a counterintuitive claim (e.g., of a miracle) that they do not necessarily believe in for the purpose of eliciting “surprise and wonder” in others as to gain their attention and respect. This account is compatible with that of the current paper: a motivation for re-transmitting counterintuitive claims may be to provoke others’ reactions to that content. Doing so may reveal whether such claims tend to be endorsed by peers. Moreover, should the counterintuitive claim be favorably received by others (perhaps as it signals a shared group membership), one may be encouraged to continually re-share it to earn their esteem.
